# Types of usual sources of care and their association with healthcare outcomes among cancer survivors: a Medical Expenditure Panel Survey (MEPS) study

**DOI:** 10.1007/s11764-022-01221-z

**Published:** 2022-06-10

**Authors:** Ambrish A. Pandit, Chenghui Li

**Affiliations:** 1 Division of Pharmaceutical Evaluation and Policy, College of Pharmacy, University of Arkansas for Medical Sciences (UAMS), Little Rock AR-72205, USA

**Keywords:** Usual source of care, Disparity, Cancer, Access, Utilization

## Abstract

**Purpose:**

To assess associations between usual source of care (USC) type and health status, healthcare access, utilization, and expenses among adult cancer survivors.

**Methods:**

This retrospective cross-sectional analysis using 2013–2018 Medical Expenditure Panel Survey included 2690 observations representing 31,953,477 adult cancer survivors who were currently experiencing cancer and reporting one of five USC types: solo practicing physician (SPP), a specific person in a non-hospital facility, a specific person in a hospital-based facility, a non-hospital facility, and a hospital-based facility. We used logistic regressions and generalized linear models to determine associations of USC type with health status, healthcare access, utilization, and expenses, adjusting for patient demographic and clinical characteristics.

**Results:**

All non-SPP USC types were associated with reporting more difficulties contacting USC by telephone during business hours (*p* < 0.05). Compared to SPP, non-hospital facility was associated with more difficulty getting needed prescriptions (OR: 1.81, *p* = 0.036) and higher annual expenses ($5225, *p* = 0.028), and hospital-based facility was associated with longer travel time (OR: 1.61, *p* = 048), more ED visits (0.13, *p* = 0.049), higher expenses ($6028, *p* = 0.014), and worse self-reported health status (OR: 1.93, *p* = 0.001), although both were more likely to open on nights/weekends (*p* < 0.05). Cancer survivors with a specific person in a hospital-based facility (vs. SPP) as USC were > twofold as likely (*p* < 0.05) to report difficulty getting needed prescriptions and contacting USC afterhours.

**Conclusions:**

Among adult cancer survivors who were currently experiencing cancer, having a non-SPP type of UCS was associated with reporting more difficulties accessing care, worse health, more ED visits, and higher total expenses.

**Implications for Cancer Survivors:**

Transitioning to SPP type of USC may result in better healthcare outcomes.

## Introduction

Equity in healthcare delivery is considered a benchmark of quality of healthcare by the Institute for Healthcare Improvement [1]. Differences in accessing care, receiving treatment, and experiencing outcomes may randomly exist in any healthcare setting. However, when these differences occur systematically, they constitute healthcare disparities [1]. Age [2], race/ethnicity [3, 4], sex [5, 6], socioeconomic status [7], or geographic location [8], or a combination of these, are the major risk factors contributing to healthcare disparities. Average or weighted average of the care quality may mask poor quality of care received by subgroups of populations.

Studies assessing access to healthcare usually adjust for whether or not a person has a usual source of care (USC) [9–11]. Lack of a USC has been associated with healthcare disparities [10]; having a USC improves timely access to medical care [12] and improves health care quality, resulting in improved health [13]. However, very few studies have compared the different types of USC and how they affect access to healthcare, healthcare utilization, and costs differently. Several South Korean studies found that having a person as a USC, as opposed to a facility, has been associated with lower odds of hospitalizations and emergency department (ED) visits [13], and better quality of care [14]. In the US healthcare setting, having a doctor’s office where physicians practice either independently or as a part of a group as the USC has been associated with improved access to healthcare as compared to hospital outpatient clinic or other clinics or health centers [15]. Moreover, person-in-facility and facility, compared to solo practicing physician (SPP) type USC, have been associated with higher odds of having an ED visit among low-income individuals [16]. While these studies focus on the general population, little is known about the associations of different types of USC with healthcare access, cost, and outcomes among cancer survivors.

Cancer survivors have unique healthcare needs manifesting from various physical, functional, and psychosocial limitations [17], and often require care from a wide range of healthcare providers including doctors, nurses, and pharmacists to manage their special needs [18]. Coordination of cancer care provided to cancer survivors by different types of providers is crucial to deliver high-quality care. A primary care physician–type USC, who could either be SPP or a specific person in a non-hospital or hospital-based facility, may be better able to act as the link between a survivor’s cancer-related and non-cancer-related care [19]. Being most familiar with a patient’s medical history, a primary care physician may also help manage comorbid conditions and/or chronic pain, provide follow-up care, and assist in palliative care [19, 20]. However, the associations of USC location (a hospital-based facility, non-hospital facility, or personal office) with healthcare outcomes are yet to be studied among cancer survivors.

Given the disease agnostic nature and the limited number of studies evaluating associations of the USC type with healthcare access and utilization, this study aimed to assess the association between the USC type and healthcare access, utilization, self-reported health status, and total healthcare expenses among adult cancer survivors who were currently experiencing cancer and reported having a USC.

## Methods

### Data sources

We used the 2013–2018 Medical Expenditure Panel Survey (MEPS) data. The MEPS is collected and maintained by the Agency for Healthcare Research and Quality [21]. Non-institutionalized individuals who have responded to the National Health Interview Survey (NHIS) form the sampling frame of MEPS household component (HC) [22]. Details of the MEPS survey process can be found elsewhere [21]. MEPS uses an overlapping panel design where data about an individual is collected over a 2-year period and data collection for 2 separate panels proceeds simultaneously over 3 rounds within a calendar year (rounds 1–3 of the new panel and rounds 3–5 of the existing panel) [22]. The MEPS-HC collects information regarding insurance coverage, income, education, employment, total healthcare utilization, and total healthcare expenses.

### Study design and cohort selection

**Fig. 1:**
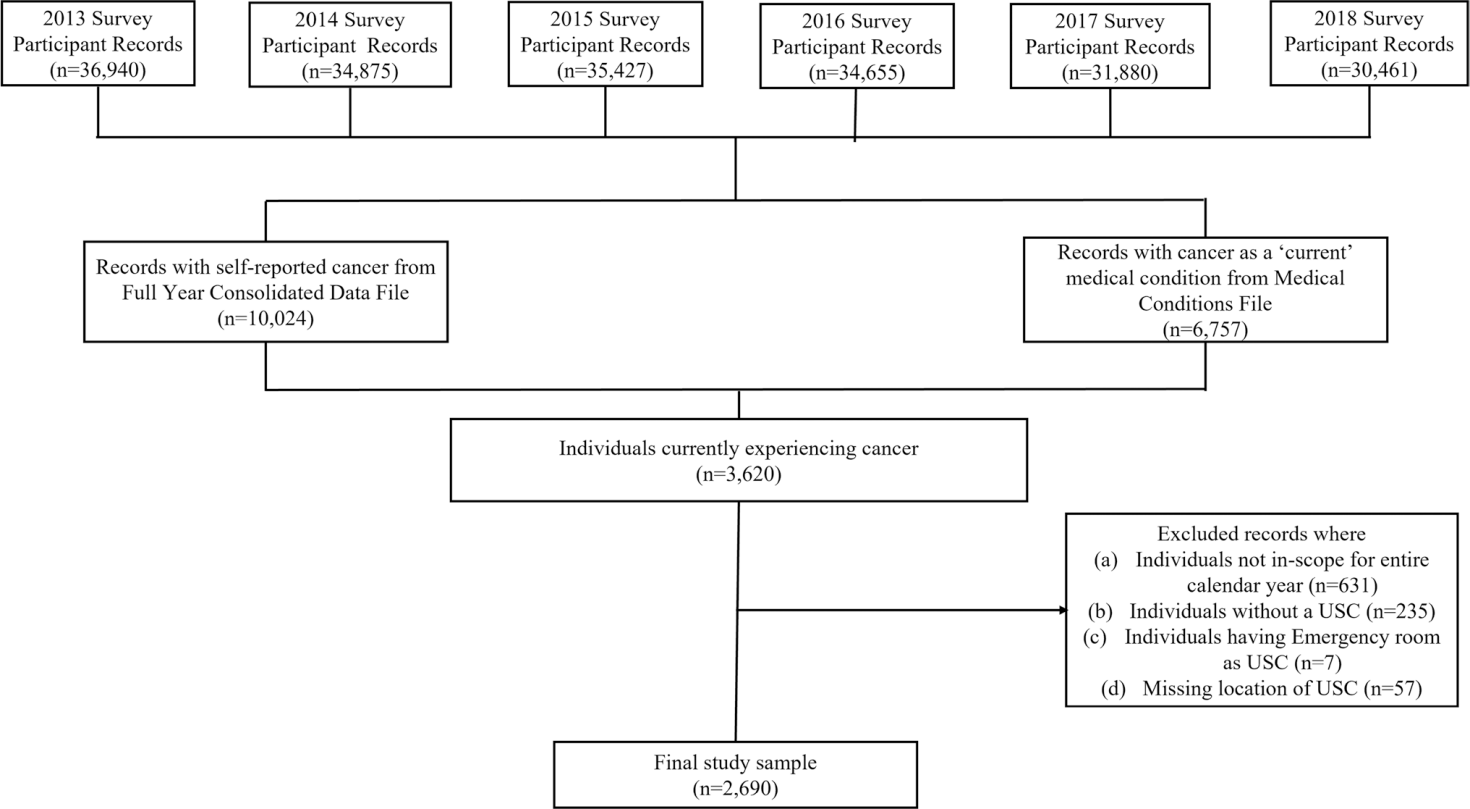
Sample selection flowchart. A *record* indicates an individual’s information for a given calendar year. “n” refers to the number of observations. A medical condition was considered “current” if it was associated with a medical event (inpatient stay, outpatient visit, office-based visit, emergency department visit, prescription medication, and home health visit) during the calendar year

This retrospective cross-sectional study consisted of adult (aged ≥ 18 years) cancer survivors who were currently experiencing cancer and reported having a USC in a calendar year. Consistent with the National Cancer Institute’s definition of a cancer survivor [23], we defined a “cancer survivor” as someone who has ever been diagnosed with cancer. In MEPS, the full-year consolidated data file includes self-reported information on whether a person has ever been diagnosed with cancer. Additionally, the medical conditions file provides information on a person’s reported “current” medical conditions that are coded using the International Classification of Diseases 9^th^ and 10^th^ revision clinical modification codes (ICD-9-CM and ICD-10-CM codes). While a *current* medical condition was defined as a “condition reported as existing for a MEPS sample person at any time during the specific data year (i.e., those identified in the conditions enumeration (CE), medical events (ME), or disability days (DD) sections of the questionnaire)” for years 2013–2017 [24], this definition was revised in 2018 to include only medical conditions that can be linked to a medical event (i.e., in the ME section) [25]. To be consistent across all years, we considered a medical condition as *current* if it was associated with a medical event (inpatient stay, outpatient visit, office-based visit, ED visit, prescription medication, and home health visit) during the calendar year. For this study, we restricted our sample to individuals self-reported as ever been diagnosed with cancer using the full-year consolidated file *and* having reported cancer as a *current* condition using the medical conditions file. Following previous studies, we did not include individuals who reported only non-melanoma skin cancer or cancer of unknown type but no other cancers [26] (see Appendix Table 1 for the diagnosis codes used to identify cancer in the medical conditions file). In addition, we restricted the study to individuals who reported having a USC. In each calendar year, we excluded records for individuals who (1) were not in-scope in all survey rounds to avoid any bias due to differential follow-up times, (2) did not report a USC, (3) reported emergency room as USC, or (4) did not report location of USC. Figure 1 provides details on cohort selection.

### Type of usual source of care

Questions regarding USC are asked once in a calendar year as a part of Access to Care (AC) section of the MEPS-HC questionnaire and are fielded in the second round (round 4/2) [27]. In MEPS, a USC is defined as a particular doctor’s office, clinic, health center, or other place that the individual usually goes to if he/she is sick or needs advice about his/her health [28]. For persons reporting a USC, MEPS determines if the provider type is a (1) facility, (2) person, or (3) person-in-facility. Furthermore, MEPS asks the provider’s location: (1) an office, (2) hospital clinic/outpatient department, or (3) a hospital emergency room. Like previous studies [29], we excluded anyone who reported a hospital emergency room as their USC. Based on the remaining provider types and location combinations, the USC type was classified into 5 mutually exclusive groups: (1) solo practicing physician (SPP) (reference group); (2) a specific person in a non-hospital facility; (3) a specific person in a hospital clinic/outpatient department (hereafter referred to as a specific person in a hospital-based facility); (4) non-hospital facility; and (5) hospital clinic/outpatient department (hereafter referred to as a hospital-based facility).

### Study outcomes

We studied the association of USC type with five types of outcomes: (a) access to USC provider; (b) delay/inability to get needed care; (c) self-reported health status; (d) healthcare utilization; and (e) total healthcare expenses. “Access to USC provider” included four measures: (1) difficulty contacting the USC provider during regular business hours over the telephone about a health problem; (2) difficulty contacting the USC provider after their regular hours in case of urgent medical needs; (3) USC open at nights/on weekends; and (4) traveling > 30 min to see USC provider. We defined the “Access to USC provider” measures as binary variables. For the first two measures with four levels, we dichotomized as follows: a person reporting “Somewhat difficult” or “Very difficult” was considered as “having difficulty” while a person reporting “Not too difficult” or “Not at all difficult” was considered “not having difficulty.” “Delay/inability to get needed care” measures included two binary measures (Yes/No) for delay/inability to get needed (1) medical care and (2) prescription medications. Self-reported health status is reported for the first (round 3/1) and last (round 5/3) rounds of each year. We used self-reported health status in the first round as a covariate to adjust for baseline health status and used the health status reported in the last round as an outcome variable. Health status as an outcome variable was defined as a binary variable: “Good” health if a person reported “excellent”/“very good”/“good” health; and “Not Good” health if reported “fair”/“poor” health. Healthcare utilization was measured as the total annual number of (1) ED visits and (2) inpatient stays, since both may be an indication of poor healthcare quality. Total annual expenses were the “sum of direct payments for care provided during the year, including out-of-pocket payments and payments by private insurance, Medicaid, Medicare, and other sources.” [28]. The sum included expenses associated with office-based physician and non-physician visits, inpatient stays, hospital outpatient visits, ED visits, prescribed medicines, home health visits, medical equipment and services, and dental and vision services [28], and was inflation-adjusted to 2018 US dollars [30].

### Covariates

For all analyses, we adjusted for the following covariates: socio-demographic variables (age, sex, race/ethnicity, family income as a percent of federal poverty level, education level, insurance), geographic region, baseline health status (self-reported health status in round 3/1), number of comorbid conditions, cancer site, calendar year, and being in remission. In MEPS, a set of medical conditions were considered priority conditions because of “their relatively high prevalence” and “generally accepted criteria for assessing appropriate clinical care.” [23]. Besides cancer, the “priority” conditions in MEPS include high blood pressure, heart disease, stroke, emphysema, chronic bronchitis, high cholesterol, diabetes, joint pain, arthritis, and asthma [23]. We counted the number of these non-cancer conditions and classified as (0, 1, or ≥ 2) following previous studies [31–33].

Remission status was not directly available in MEPS. To determine whether a survivor was being actively treated or in remission, we developed an algorithm as follows. We first examined the self-reported visit categories for outpatient and office-based visits from respective event files for all the persons in the study sample. If a visit was categorized as “Diagnosis or Treatment” or “Follow-up or Post-operative visit,” then we considered the event was for active treatment of cancer. Outpatient and office visits for all other reasons (“General checkup,” “Emergency (e.g., accident or injury),” “Psychotherapy/Mental Health Counselling,” “Immunizations or shots,” “Vision Exam,” “Pregnancy-related (including prenatal/delivery),” “Well Child Exam,” “Laser Eye Surgery,” and “Other”) were considered not for active cancer treatment. Since this variable is only available for outpatient or office-based visits, we used the following algorithm to identify active cancer treatment for other healthcare events. If a home health visit, inpatient stay, or an ED visit was reported to be related to cancer, we considered the visit to be for cancer treatment. If a prescription was for drugs in “antineoplastics,” “antineoplastic hormones,” or “topical antineoplastics” therapeutic classes, we considered that the prescription was for cancer treatment. All persons who did not have any medical event indicating they were being actively treated for cancer by our aforementioned definition were considered to be in remission in that year. Appendix Table 2 provides further description of the covariates.

### Statistical analysis

We assessed associations of each of the outcomes with USC type adjusting for the covariates. Logistic regressions were used for six binary outcomes: (1) delay/inability to get needed medical care; (2) delay/inability to get needed prescription medications; (3) difficulty contacting the USC provider during regular business hours over the telephone about a health problem; (4) difficulty contacting the USC provider after their regular hours in case of urgent medical needs; (5) USC open at nights/on weekends; and (6) traveling > 30 min to see USC provider. Generalized linear models (GLMs) with a negative binomial distribution and a log link were used to analyze count outcomes: (1) total ED visits and (2) total inpatient stays since these outcomes had distributions that were over-dispersed and right skewed with heavy kurtosis. GLM with a gamma distribution and a log link was used to analyze total expenses since the expenses were right skewed with heavy kurtosis.

We used SAS v.9.4 and STATA v.16.1 to perform statistical analysis. STATA svy commands were used for all bivariate and multivariate analyses to adjust for complex survey design and obtain nationally representative estimates of the non-institutionalized population and standard errors were adjusted for survey clustering using Taylor series linearization [34]. Since the MEPS is de-identified public-use data, no IRB approval was needed.

## Results

### Study cohort and demographics

After applying inclusion and exclusion, the study sample included 2690 observations representing 31,953,477 cancer survivors over the study period. Table 1 compares the demographic and clinical characteristics of cancer survivors by USC type. In the unweighted sample, 24.4% had SPP, 20.1% had non-hospital facility, 16.1% had hospital-based facility, 27.9% had a specific person in a non-hospital facility, and 11.5% had a specific person in a hospital-based facility as their USC. The study sample had 68.3% non-Hispanic whites, 13.8% non-Hispanic blacks, 12.6% Hispanics, and 5.3% belonging to other races. About 60% of the cohort either had private insurance or were uninsured, aged ≥ 65 years, and had ≥ 2 comorbidities. Moreover, about 30% of the cohort reported fair or poor health in the first round (round 1/3), 67.9% having high income and 84.8% having a high school diploma or higher education. The study sample had a slightly higher proportion of females as compared to males. About one-fourth of the survivors were in remission. There was no difference in remission status by USC type.

### Health status and access to healthcare

Table 2 provides adjusted odds ratios (ORs) and 95% confidence intervals (CIs) for associations of self-reported health and healthcare access outcomes with USC type. When compared to survivors with SPP-type USC, survivors with hospital-based facility–type USC reported higher odds (OR: 1.93, 95% CI: 1.32–2.83; *p* = 0.001) of having “Not good” health in round 5/3. Compared to SPP, survivors with all other types of USC had significantly higher odds of facing difficulty in contacting USC during regular business hours over the telephone about a health problem, with ORs ranging from 1.66 (95% CI: 1.09–2.54; *p* = 0.019) for a non-hospital facility to 2.08 (95% CI: 1.27–3.40; *p* = 0.004) for a specific person in a hospital-based facility–type USC. Moreover, people having a specific person in a hospital-based facility (compared to SPP) type USC also faced more difficulty contacting the USC during afterhours in case of urgent medical needs (OR: 2.07, 95% CI: 1.24–3.47; *p* = 0.006) and accessing needed prescription medications (OR: 2.28, 95% CI: 1.10–4.73; *p* = 0.028). People with non-hospital facility (compared to those with SPP) type USC reported higher odds of facing delay and/or inability in accessing needed prescription medications (OR: 1.81, 95% CI: 1.04–3.16; *p* = 0.036). On the other hand, survivors with non-hospital facility (OR: 3.81, 95% CI: 2.57–5.66; *p* < 0.001) and hospital-based facility (OR: 4.19, 95% CI: 2.72–6.44; *p* < 0.001) type USC reported higher odds of their USC being open at nights/weekends compared to survivors with SPP-type USC. Only survivors with hospital-based facility–type USC reported significantly higher odds (OR: 1.61, 95% CI: 1.00–2.58; *p* = 0.048) of travelling > 30 min to see USC provider. We did not find any significant differences by USC type in reporting delay/inability to get needed medical care.

### Annual healthcare utilization and total expenses

Table 3 provides adjusted predicted means and differences in predicted means and 95% CIs for inpatient stays, ED visits, and total expenses. Survivors with hospital-based facility–type USC reported on average more ED visits annually (0.13 [95% CI: 0.00–0.26]; *p* = 0.049) and higher average annual total expenses ($6028 [95% CI: $1235–$10,822]; *p* = 0.014) as compared to survivors with SPP-type USC. Additionally, survivors with non-hospital facility–type USC also reported incurring higher average annual total expenses ($5225 [95% CI: $557–$9892]; *p* = 0.028) as compared to survivors with SPP-type USC. We did not find any significant association between USC type and the number of inpatient stays.

## Discussion

In this nationally representative sample of adult cancer survivors with a non-ED USC type who were currently experiencing cancer, we found that different USC types when compared to SPP were associated with a greater likelihood of reporting difficulty in accessing care, “Not good” health, more ED visits, and higher total expenses.

Compared to SPP, survivors with all other USC types reported more difficulties contacting their USC providers over telephone during regular business hours. The ability to consult with providers regarding health problems through telecommunication technologies such as a telephone is a key aspect of telemedicine [35]. Telemedicine has been popular among cancer survivors who do not have easy access to a healthcare facility or must travel a long distance for medical appointment [36]. Besides being highly useful in delivering care to populations that have limited access to healthcare, telemedicine has been shown to be at par with in-person care [37]. Moreover, telemedicine also helps decrease healthcare costs [37]. Post-operative telemedicine provided to cancer survivors may also reduce ED visits [38]. Thus, difficulty in contacting USC about health problems by telephone during regular business hours would augment healthcare disparities among cancer survivors with non-SPP types of USC. On the other hand, having a non-hospital or a hospital-based facility–type USC may offer additional access during nights or weekends. We found that non-hospital facility and hospital-based facility types of USC were associated with significantly higher odds of being open during nights or weekends. Survivors with facility type of USC may have an option to see any provider available during nights or weekends, but continuity of care may be jeopardized.

Consistent with previous findings [13], hospital-based facility (compared to SPP) type USC was associated with a significantly higher mean number of ED visits. Acute healthcare utilization such as ED visits is often an indication of poor access to primary care, poor care coordination, and poor choices on the part of beneficiaries [39]. Given the high costs associated with ED visits, avoiding preventable ED visits would significantly help reduce total healthcare costs [40]. The high mean number of ED visits partially explains the significantly higher mean total healthcare expenses for hospital-based facility as compared to person-facility–type USC. Moreover, survivors with non-hospital facility compared to SPP-type USC also reported significantly higher mean total healthcare expenses. Healthcare services provided by USC providers located in a hospital facility (hospital-based facility/a specific person in a hospital-based facility) would include facility charges, in addition to charges for professional services, while USC providers in offices outside of hospital would only charge for professional services. Such additional charges would also increase the total healthcare expenses for cancer survivors who seek care through USC types having hospital-based locations.

Having a SPP-type USC indicates that the patient would most often interact with the same provider. Such interactions may eventually lead to developing strong patient-provider relationship [41]. Survivors who have an ongoing relationship with their provider are likely to develop trust in their provider and receive more personalized care, which may help in better adherence to provider’s advice and timely follow-up [42]. A high likelihood of seeing a different provider during health service encounters may lead to lack of care coordination, polypharmacy, higher chances of medical errors, poorly controlled symptoms, and higher medical costs [43]. While medical errors and poorly controlled symptoms may increase odds of a person reporting “Not Good” health, polypharmacy, lack of adherence to medical advice, and high healthcare costs may also explain higher odds of delay/inability to get needed prescription medications.

Our study findings show that different non-SPP types of USC were associated with worse healthcare access, poorer health outcome, and higher healthcare expenses. These findings highlight the importance of having a SPP-type USC among cancer survivors. With the ongoing national trend of physician practice mergers and acquisitions, access to SPPs may continue to decrease. A Physicians Advocacy Institute (PAI) report found a steep rise in the number of hospital-acquired physician practices over a period from mid-2012 through January 2018 [44]. The proportion of all the physicians in the USA who were employed by hospitals jumped from 25% in 2012 to 44% in early 2018, marking a 76% rise [44]. However, whether this consolidation improves patient care or lowers healthcare costs has been questioned. Consistent with our findings, research conducted by Avalere and released by PAI found that between 2012 and 2015, Medicare costs and financial responsibility of Medicare beneficiaries increased by $3.1 billion and $411 million, respectively, when using select cardiology, orthopedic, and gastroenterology services performed by hospital-employed physicians compared to if they were performed in independent physicians’ offices [45]. Moreover, routine services such as cardiac imaging, colonoscopy, and evaluation and management consistently cost higher when performed in hospital outpatient department as opposed to a physician’s office [45]. Larger studies are warranted to confirm our findings about poor healthcare outcomes among cancer survivors with non-SPP types of USC.

Several limitations of this study are worth noting. MEPS does not provide cancer-related clinical variables such as age at diagnosis, cancer stage, tumor grade, and other histological characteristics. Remission status was not directly available in MEPS. The variable for being in remission was derived from (1) self-reported reasons for office-based and outpatient visits; (2) inpatient, ED, or home health visits linked to cancer condition; or (3) antineoplastic prescriptions use, which may not accurately represent the clinical remission status. Moreover, we do not have detailed information regarding the type of treatment such as radiation or systemic chemotherapy use and therefore could not determine how patterns of cancer care differ by USC type. Despite adjusting our regression models for key socio-demographic characteristics, unmeasured variables such as health literacy, attitudes, and beliefs regarding healthcare resource utilization could potentially confound healthcare utilization. In MEPS, information about the USC is asked only in the second round (4/2), but a person may change USC type over the year.

In this nationally representative study of cancer survivors with a USC who was currently experiencing cancer, having any non-SPP (vs. SPP) type of USC was found to be associated with reporting more difficulties accessing care, worse health, more ED visits, and higher total expenses. Further research is warranted to replicate and extend findings of this study to better understand key factors contributing to these differences among cancer survivors.

## Supplementary Material

Supplementary Materials

## Figures and Tables

**Table 1:** Demographic characteristics of adult cancer survivors reporting one of the five mutually exclusive types of USC in 2013–2018 Medical Expenditure Panel Survey (MEPS) data

Variables	Weighted total (*n* = 31,953,477)	Unweighted number of observations (%) (*n* = 2690)					*p*-value
		Non-hospital facility (*n* = 540)	Hospital-based facility (*n* = 433)	Solo practicing physician (*n* = 656)	A specific person in a non-hospital facility (*n* = 751)	A specific person in a hospital-based facility (*n* = 310)	
	% (SE)	*n* (%)	*n* (%)	*n* (%)	*n* (%)	*n* (%)	

Race							< 0.001
Non-Hispanic White	81.4 (1.1)	372 (68.89)	232 (53.58)	444 (67.68)	583 (77.63)	207 (66.77)	
Non-Hispanic Black	7.7 (0.7)	71 (13.15)	74 (17.09)	84 (12.80)	95 (12.65)	48 (15.48)	
Other	11.0 (0.8)	35 (6.48)	24 (5.54)	42 (6.40)	25 (3.33)	16 (5.16)	
Sex							0.130
Female	54.2 (1.4)	308 (57.04)	223 (51.50)	353 (53.81)	424 (56.46)	154 (49.68)	
Male	45.8 (1.4)	232 (42.96)	210 (48.50)	303 (46.19)	327 (43.54)	156 (50.32)	
Region							< 0.001
Northeast	20.9 (1.5)	98 (18.15)	53 (12.24)	171 (26.07)	180 (23.97)	37 (11.94)	
Midwest	22.8 (1.6)	104 (19.26)	102 (23.56)	92 (14.02)	170 (22.64)	92 (29.68)	
South	34.8 (1.6)	218 (40.37)	132 (30.48)	273 (41.62)	276 (36.75)	88 (28.39)	
West	21.5 (1.3)	120 (22.22)	146 (33.72)	120 (18.29)	125 (16.64)	93 (30.00)	
Age categories							< 0.001
18–49 years	10.6 (0.8)	85 (15.74)	91 (21.01)	61 (9.30)	73 (9.72)	< 30 (< 9.68)	
50–64 years	27.9 (1.3)	156 (28.89)	128 (29.56)	190 (28.96)	179 (23.83)	> 79 (> 25.48)	
65–74 years	31.5 (1.2)	166 (30.74)	120 (27.71)	203 (30.95)	261 (34.75)	109 (35.16)	
≥ 75 years	30.0 (1.4)	133 (24.63)	94 (21.71)	202 (30.79)	238 (31.69)	92 (29.68)	
Calendar year							< 0.001
2013	12.2 (0.9)	78 (14.44)	48 (11.09)	85 (12.96)	79 (10.52)	< 30 (< 9.68)	
2014	16.4 (1.0)	84 (15.56)	58 (13.39)	81 (12.35)	110 (14.65)	36 (11.61)	
2015	10.6 (0.7)	67 (12.41)	51 (11.78)	76 (11.59)	61 (8.12)	< 30 (< 9.68)	
2016	21.0 (1.0)	119 (22.04)	114 (26.33)	147 (22.41)	129 (17.18)	50 (16.13)	
2017	20.7 (0.9)	143 (26.48)	109 (25.17)	123 (18.75)	142 (18.91)	61 (19.68)	
2018	19.1 (1.0)	49 (9.07)	53 (12.24)	144 (21.95)	230 (30.63)	120 (38.71)	
Education							< 0.001
High school diploma or higher	89.6 (0.7)	464 (85.93)	336 (77.60)	554 (84.45)	657 (87.48)	271 (87.42)	
Less than high school diploma/Unknown	10.4 (0.7)	76 (14.07)	97 (22.40)	102 (15.55)	94 (12.52)	39 (12.58)	
Family income							< 0.001
High (≥ 200% of FPL)	74.8 (1.2)	362 (67.04)	256 (59.12)	461 (70.27)	546 (72.70)	201 (64.84)	
Low (< 200% of FPL)	25.2 (1.2)	178 (32.96)	177 (40.88)	195 (29.73)	205 (27.30)	109 (35.16)	
Insurance (throughout the year)							0.028
Any private/uninsured	67.3 (1.5)	319 (59.07)	243 (56.12)	413 (62.96)	474 (63.12)	172 (55.48)	
Public only	32.7 (1.5)	221 (40.93)	190 (43.88)	243 (37.04)	277 (36.88)	138 (44.52)	
Comorbidities							0.384
0	14.2 (0.9)	79 (14.63)	81 (18.71)	107 (16.31)	114 (15.18)	42 (13.55)	
1	24.9 (1.2)	128 (23.70)	111 (25.64)	163 (24.85)	167 (22.24)	77 (24.84)	
≥ 2	60.9 (1.4)	333 (61.67)	241 (55.66)	386 (58.84)	470 (62.58)	191 (61.61)	
Being in remission							0.664
No	73.4 (1.2)	399 (73.89)	320 (73.90)	485 (73.93)	539 (71.77)	236 (76.13)	
Yes	26.6 (1.2)	141 (26.11)	113 (26.10)	171 (26.07)	212 (28.23)	74 (23.87)	
Health status round 3/1							0.028
Excellent	13.4 (0.9)	57 (10.56)	40 (9.24)	78 (11.89)	110 (14.65)	37 (11.94)	
Very good	28.4 (1.1)	143 (26.48)	98 (22.63)	170 (25.91)	199 (26.50)	79 (25.48)	
Good	31.5 (1.1)	172 (31.85)	137 (31.64)	216 (32.93)	252 (33.56)	102 (32.90)	
Fair	18.5 (1.0)	105 (19.44)	114 (26.33)	125 (19.05)	135 (17.98)	> 62 (> 20.00)	
Poor	8.2 (0.7)	63 (11.67)	44 (10.16)	67 (10.21)	55 (7.32)	< 30 (9.68)	
Cancer site							
Breast	21.7 (1.1)	105 (19.44)	94 (21.71)	129 (19.66)	168 (22.37)	78 (25.16)	0.243
Prostate	17.4 (1.0)	85 (15.74)	86 (19.86)	120 (18.29)	139 (18.51)	63 (20.32)	0.415
Melanoma^#^	12.7 (0.9)	57 (10.56)	36 (8.31)	69 (10.52)	84 (11.19)	38 (12.26)	0.467
Any other cancer except breast cancer, prostate cancer, and melanoma	49.8 (1.4)	300 (55.56)	221 (51.04)	345 (52.59)	365 (48.60)	137 (44.19)	0.029

*USC*, usual source of care; *FPL*, Federal poverty line; *SE*, standard error

# Not mutually exclusive of breast cancer and prostate cancer

Note: Weighted total percentage and standard error values are for the US civilian non-institutionalized population of adult cancer survivors currently experiencing cancer and having a usual source of care during 2013–2018. Percentages may not add up to 100.0% due to rounding. *p*-values based on chi-square tests. Cell sizes < 30 have been collapsed

**Table 2:** Adjusted odds ratios and 95% confidence intervals for association of USC type with self-reported health and healthcare access outcomes^#^

Variable (reference category), weighted *n*	Non-hospital facility vs. SPP	Hospital-based facility vs. SPP	A specific person in a non-hospital facility vs. SPP	A specific person in a hospital-based facility vs. SPP
	AOR (95% CI)	AOR (95% CI)	AOR (95% CI)	AOR (95% CI)

Health (ref = Good health), *n* = 31,953,477	1.15 (0.80–1.65)	**1.93 (1.32–2.83)** **	1.12 (0.81–1.55)	0.95 (0.62–1.47)
Delay/forgone access to medical care (ref = No.), *n* = 31,874,313	1.14 (0.62–2.12)	1.15 (0.60–2.21)	1.11 (0.64–1.92)	1.29 (0.65–2.56)
Delay/forgone access to prescription medications (ref = No.), *n* = 31,865,340	**1.81 (1.04–3.16)** *	1.54 (0.83–2.84)	1.68 (0.98–2.88)	**2.28 (1.10–4.73)** *
Difficulty contacting USC...				
...During afterhours for urgent medical needs (ref = No.), *n* = 18,309,466	1.31 (0.85–2.00)	1.15 (0.75–1.76)	1.27 (0.86–1.87)	**2.07 (1.24–3.47)** **
...During regular business hours by phone about a health problem (ref = No.), *n* = 30,580,570	**1.66 (1.09–2.54)** *	**1.91 (1.17–3.12)** *	**1.71 (1.08–2.71)** *	**2.08 (1.27–3.40)** **
USC open at nights/weekends (ref = No.), *n* = 28,305,377	**3.81 (2.57–2.57)** ***	**4.19 (2.72–6.44)** ***	1.19 (0.83–1.71)	0.93 (0.54–1.59)
Travel time to USC > 30 min (ref = No.), *n* = 31,890,229	0.80 (0.46–1.38)	**1.61 (1.00–2.58)** *	0.90 (0.55–1.49)	1.30 (0.76–2.24)

* < 0.05

** < 0.01

*** < 0.001

*USC*, usual source of care; *SPP*, solo practicing physician; *AOR*, adjusted odds ratio; CI, confidence interval

# Regression models adjusted for age, sex, race/ethnicity, family income as a % of federal poverty level, education level, insurance, geographic region, self-reported health status in round 3/1, number of comorbid conditions, cancer site, calendar year, and being in remission

Note: Sample size and regression model estimates are for the US civilian non-institutionalized population of adult cancer survivors currently experiencing cancer and having a usual source of care during 2013–2018

**Table 3:** Adjusted predicted means and differences in predict means from covariate-adjusted generalized linear regression models for association of USC type with annual number of inpatient stays, ED visits, and total expenses^#^

Variable and USC type, weighted *n*	Adjusted predicted mean	95% CI for predicted mean	Difference in predicted mean from SPP	95% CI for difference	*p*-value

**Number of annual inpatient stays, *n* = 31,953,477**					
Solo practicing physician (SPP), *n* = 8,003,366	0.29	(0.22–0.36)			
Non-hospital facility, *n* = 6,989,757	0.33	(0.25–0.41)	0.03	(−0.08 to 0.14)	0.567
Hospital-based facility, *n* = 4,286,642	0.36	(0.25–0.47)	0.08	(−0.06 to 0.21)	0.255
A specific person in a non-hospital facility, *n* = 9,146,547	0.31	(0.25–0.37)	0.02	(−0.06 to 0.10)	0.602
A specific person in a hospital-based facility, *n* = 3,527,165	0.26	(0.17–0.36)	−0.02	(−0.14 to 0.09)	0.693
**Number of annual ED visits, *n* = 31,953,477**					
SPP, *n* = 8,003,366	0.32	(0.26–0.38)			
Non-hospital facility, *n* = 6,989,757	0.32	(0.25–0.38)	−0.01	(−0.10 to 0.08)	0.848
Hospital-based facility, *n* = 4,286,642	0.45	(0.34–0.56)	**0.13** *	**(0.00–0.26)**	**0.049**
A specific person in a non-hospital facility, *n* = 9,146,547	0.39	(0.32–0.46)	0.06	(−0.03 to 0.15)	0.158
A specific person in a hospital-based facility, *n* = 3,527,165	0.37	(0.26–0.49)	0.05	(−0.08 to 0.17)	0.450
**Annual total expenses (in 2018 US dollars), *n* = 31,953,477**					
SPP, *n* = 8,003,366	$18,320	($15,639–$21,000)			
Non-hospital facility, *n* = 6,989,757	$23,743	($19,781–$27,706)	**$5225** *	**($557–$9892)**	**0.028**
Hospital-based facility, *n* = 4,286,642	$23,983	($19,724–$28,243)	**$6028** *	**($1235–$10,822)**	**0.014**
A specific person in a non-hospital facility, *n* = 9,146,547	$21,392	($18,528–$24,255)	$3192	((–)$402 to $6,786)	0.082
A specific person in a hospital-based facility, *n* = 3,527,165	$20,100	($15,023–$25,176)	$2881	((–)$3079 to $8841)	0.342

* < 0.05

** < 0.01

*** < 0.001

*USC*, usual source of care; *ED*, emergency department; *CI*, confidence interval; *SPP*, solo practicing physician

Bolded values are significant at *p* < 0.05

# Regression models adjusted for age, sex, race/ethnicity, family income as a % of federal poverty level, education level, insurance, geographic region, self-reported health status in round 3/1, number of comorbid conditions, cancer site, calendar year, and being in remission

Note: Sample size and regression model estimates are for the US civilian non-institutionalized population of adult cancer survivors currently experiencing cancer and having a usual source of care during 2013–2018
