# Supplementary material for: Types of usual sources of care and their association with healthcare outcomes among cancer survivors: a Medical Expenditure Panel Survey (MEPS) study
Source: J Cancer Surviv. 2022 Jun 10;17(3):748–58. doi: 10.1007/s11764-022-01221-z (PMC10016387; doi:10.1007/s11764-022-01221-z)
Supplement: Supplementary file 1 — Supplementary file1 (DOCX 20 KB) [file 11764_2022_1221_MOESM1_ESM.docx]

**Table 1: International Classification of Diseases (ICD) Clinical modification (CM) revision 9 and 10 codes**

| **Type of diagnosis code** | **Code** |
| --- | --- |
| **ICD-9-CM** | 141, 142, 143, 144, 145, 146, 147, 148, 149, 150,  151, 152, 153, 154, 155, 156, 157, 158, 159, 160, 161, 162, 163, 164, 165,  166, 167, 168, 169, 170, 171, 172, 173, 174, 175, 176, 177, 178, 179, 180,  181, 182, 183, 184, 185, 186, 187, 188, 189, 190, 191, 192, 193, 194, 195,  196, 197, 198, 199, 200, 201, 202, 203, 204, 205, 206, 207, 208, 209, 210,  211, 212, 213, 214, 215, 216, 217, 218, 219, 220, 221, 222, 223, 224, 225,  226, 227, 228, 229, 230, 231, 232, 233, 234, 235, 236, 237, 238, 239 |
| **ICD-10-CM** | C00, C01, C02, C03, C04, C05, C06, C07, C08, C09, C10,  C11, C12, C13, C14, C15, C16, C17, C18, C19, C20  C20, C21, C22, C23, C24, C25, C26, C27, C28, C29, C30,  C31, C32, C33, C34, C35, C36, C37, C38, C39, C40,  C41, C42, C43, C44, C45, C46, C47, C48, C49, C50,  C51, C52, C53, C54, C55, C56, C57, C58, C59, C60,  C61, C62, C63, C64, C65, C66, C67, C68, C69, C70,  C71, C72, C73, C74, C75, C76, C77, C78, C79, C80,  C81, C82, C83, C84, C85, C86, C87, C88, C89, C90  C91, C92, C93, C94, C95, C96, C7A, C7B,  D00, D01, D02, D03, D04, D05, D06, D07, D08, D09, D10,  D11, D12, D13, D14, D15, D16, D17, D18, D19, D20,  D21, D22, D23, D24, D25, D26, D27, D28, D29, D30,  D31, D32, D33, D34, D35, D36, D37, D38, D39, D40,  D41, D42, D43, D44, D45, D46, D47, D48, D49, D3A |

**Table 2: Covariates, their description and proposed role in multivariate regression models**

| **Variable** | **Description** | **Role** |
| --- | --- | --- |
| Race | 1. **Non-Hispanic White (reference category)** 2. Non-Hispanic Black 3. Other races | Confounder |
| Sex | 1. **Female (reference category)** 2. Male | Confounder |
| Region | 1. **Northeast (reference category)** 2. Midwest 3. South 4. West | Confounder |
| Age | 1. **18-49 years (reference category)** 2. 50-64 years 3. 65-74 years 4. ≥75 years | Confounder |
| Calendar year | 1. **2013 (reference category)** 2. 2014 3. 2015 4. 2016 5. 2017 6. 2018 | Covariate |
| Education | 1. **High school diploma or higher: At least high school diploma or its equivalent or higher (reference category)** 2. Less than high school diploma/Missing: Less than high school diploma or missing value | Confounder |
| Family income | 1. **High income: ≥200% of federal poverty line (reference category)** 2. Low income i.e. <200% of federal poverty line | Confounder |
| Insurance | 1. **Any private/Uninsured: Person had any private insurance or was uninsured throughout the year (reference category)** 2. Public only | Confounder |
| Comorbidities | 1. **0: No comorbidities (reference category)** 2. 1: 1 comorbidity 3. ≥2: ≥2 comorbidities | Confounder |
| Being in remission | 1. **Yes: A person did not receive any cancer-related treatment/follow-up care throughout the year (reference category)** 2. No: A person is received treatment/follow-up care for cancer during the year | Confounder |
| Health status round 3/1 | 1. **Excellent (reference category)** 2. Very good 3. Good 4. Fair 5. Poor | Confounder |
| Cancer site^#^ |  |  |
| Breast | 1. **No** 2. Yes | Covariate |
| Prostate | 1. **No** 2. Yes | Covariate |
| Melanoma^#^ | 1. **No** 2. Yes | Covariate |
| Any other cancer except breast cancer, prostate cancer and melanoma | 1. **No** 2. Yes | Covariate |
