# Supplementary material for: Types of usual sources of care and their association with healthcare outcomes among cancer survivors: a Medical Expenditure Panel Survey (MEPS) study
Source: J Cancer Surviv. Author manuscript; Available in PMC 2023 Jun 1. (PMC10016387; doi:10.1007/s11764-022-01221-z)
Supplement: Supplementary file 1 [file NIHMS1877029-supplement-Supplementary_Materials.pdf]

Table 1: International Classification of Diseases (ICD) Clinical modification (CM) revision 9 and 10 codes

Type of diagnosis code	Code
**ICD-9-CM**	141, 142, 143, 144, 145, 146, 147, 148, 149, 150, 151, 152, 153, 154, 155, 156, 157, 158, 159, 160, 161, 162, 163, 164, 165,<br>166, 167, 168, 169, 170, 171, 172, 173, 174, 175, 176, 177, 178, 179, 180,<br>181, 182, 183, 184, 185, 186, 187, 188, 189, 190, 191, 192, 193, 194, 195,<br>196, 197, 198, 199, 200, 201, 202, 203, 204, 205, 206, 207, 208, 209, 210,<br>211, 212, 213, 214, 215, 216, 217, 218, 219, 220, 221, 222, 223, 224, 225,<br>226, 227, 228, 229, 230, 231, 232, 233, 234, 235, 236, 237, 238, 239
**ICD-10-CM**	C00, C01, C02, C03, C04, C05, C06, C07, C08, C09, C10,<br>C11, C12, C13, C14, C15, C16, C17, C18, C19, C20<br>C20, C21, C22, C23, C24, C25, C26, C27, C28, C29, C30,<br>C31, C32, C33, C34, C35, C36, C37, C38, C39, C40,<br>C41, C42, C43, C44, C45, C46, C47, C48, C49, C50,<br>C51, C52, C53, C54, C55, C56, C57, C58, C59, C60,<br>C61, C62, C63, C64, C65, C66, C67, C68, C69, C70,<br>C71, C72, C73, C74, C75, C76, C77, C78, C79, C80,<br>C81, C82, C83, C84, C85, C86, C87, C88, C89, C90<br>C91, C92, C93, C94, C95, C96, C7A, C7B,<br>D00, D01, D02, D03, D04, D05, D06, D07, D08, D09, D10,<br>D11, D12, D13, D14, D15, D16, D17, D18, D19, D20,<br>D21, D22, D23, D24, D25, D26, D27, D28, D29, D30,<br>D31, D32, D33, D34, D35, D36, D37, D38, D39, D40,<br>D41, D42, D43, D44, D45, D46, D47, D48, D49, D3A

Table 2: Covariates, their description and proposed role in multivariate regression models

Variable	Description	Role
Race	(a) **Non-Hispanic White (reference category)**<br>(b) Non-Hispanic Black<br>(c) Other races	Confounder
Sex	(a) **Female (reference category)**<br>(b) Male	Confounder
Region	(a) **Northeast (reference category)**<br>(b) Midwest<br>(c) South<br>(d) West	Confounder
Age	(a) **18-49 years (reference category)**<br>(b) 50-64 years<br>(c) 65-74 years<br>(d) ≥75 years	Confounder
Calendar year	(a) **2013 (reference category)**<br>(b) 2014<br>(c) 2015<br>(d) 2016<br>(e) 2017<br>(f) 2018	Covariate
Education	(a) **High school diploma or higher: At least high school diploma or its equivalent or higher (reference category)**<br>(b) Less than high school diploma/Missing: Less than high school diploma or missing value	Confounder
Family income	(a) **High income: ≥200% of federal poverty line (reference category)**<br>(b) Low income i.e. <200% of federal poverty line	Confounder
Insurance	(a) **Any private/Uninsured: Person had any private insurance or was uninsured throughout the year (reference category)**<br>(b) Public only	Confounder

Variable	Description	Role
Comorbidities	(a) **0: No comorbidities (reference category)**<br>(b) 1: 1 comorbidity<br>(c) ≥2: ≥2 comorbidities	Confounder
Being in remission	(a) **Yes: A person did not receive any cancer-related treatment/follow-up care throughout the year (reference category)**<br>(b) No: A person is received treatment/follow-up care for cancer during the year	Confounder
Health status round 3/1	(a) **Excellent (reference category)**<br>(b) Very good<br>(c) Good<br>(d) Fair<br>(e) Poor	Confounder
Cancer site#		
Breast	(a) **No**<br>(b) Yes	Covariate
Prostate	(a) **No**<br>(b) Yes	Covariate
Melanoma#	(a) **No**<br>(b) Yes	Covariate
Any other cancer except breast cancer, prostate cancer and melanoma	(a) **No**<br>(b) Yes	Covariate
